# The Mediating Role of Sleep Disturbance on the Association Between Stress and Self-Rated Health Among Chinese and Korean Immigrant Americans

**DOI:** 10.5888/pcd20.220241

**Published:** 2023-01-26

**Authors:** Brittany N. Morey, Soomin Ryu, Yuxi Shi, Sunmin Lee

**Affiliations:** 1 Department of Health, Society, and Behavior, Program in Public Health, University of California, Irvine; 2 Department of Epidemiology, School of Public Health, University of Michigan, Ann Arbor; 3 Department of Medicine, School of Medicine, University of California, Irvine

## Abstract

**Introduction:**

Disturbed sleep may be a factor that links stress with poor health, especially among groups experiencing high levels of stress caused by racial and ethnic minority and nativity status. The objective of this study was to describe the potential mediating role of sleep disturbance in the associations between various types of stress and self-rated health among Chinese and Korean Americans.

**Methods:**

Our cross-sectional study consisted of 400 Chinese and Korean immigrants aged 50 to 75 years recruited from August 2018 through June 2020 from physicians’ clinics in the Baltimore–Washington, DC, metropolitan area. We used the Patient Reported Outcomes Measurement Information System (PROMIS) short-form questionnaire to measure sleep disturbance. Linear regression analyses examined associations between 3 types of stress (acculturative stress, perceived stress, and distress) and self-rated health, accounting for demographic, socioeconomic, and health insurance factors. The Karlson–Holm–Breen method was used to estimate the total and direct effects of stresses on self-rated health and the indirect effects of stresses on health through sleep disturbance.

**Results:**

Greater acculturative stress (β = 0.08; 95% CI, 0.01–0.14), perceived stress (β = 0.05; 95% CI, 0.03–0.08), and distress (β = 0.09; 95% CI, 0.05–0.13) were all associated with poorer self-rated health. Sleep disturbance was a partial mediator, with sleep disturbance accounting for 21.7%, 14.9%, and 18.7% of the associations between acculturative stress, perceived stress, and distress and self-rated health, respectively.

**Conclusion:**

Because sleep disturbance partially mediates the associations between stress and poor self-rated health, future interventions and research may consider mitigating sleep disturbances and stress among racial and ethnic minority populations to address health disparities.

**Summary**

**What is already known on this topic?**

Racial and ethnic minority populations in the US experience heightened stress, which leads to racial and ethnic disparities in chronic disease.

**What is added by this report?**

Sleep disturbance is a crucial mediator, explaining part of the associations between stress and poor health among Chinese and Korean immigrants in the US.

**What are the implications for public health practice?**

Improving sleep health can help to decrease the effects of heightened stress on chronic disease outcomes among racial and ethnic minority populations.

**MEDSCAPE CME**

In support of improving patient care, this activity has been planned and implemented by Medscape, LLC and *Preventing Chronic Disease*. Medscape, LLC is jointly accredited with commendation by the Accreditation Council for Continuing Medical Education (ACCME), the Accreditation Council for Pharmacy Education (ACPE), and the American Nurses Credentialing Center (ANCC), to provide continuing education for the healthcare team.

Medscape, LLC designates this Journal-based CME activity for a maximum of 1.0 AMA PRA Category 1 Credit(s)™. Physicians should claim only the credit commensurate with the extent of their participation in the activity.

Successful completion of this CME activity, which includes participation in the evaluation component, enables the participant to earn up to 1.0 MOC points in the American Board of Internal Medicine’s (ABIM) Maintenance of Certification (MOC) program. Participants will earn MOC points equivalent to the amount of CME credits claimed for the activity. It is the CME activity provider’s responsibility to submit participant completion information to ACCME for the purpose of granting ABIM MOC credit.

**Release date: **January 26, 2023; **Expiration date:** January 26, 2024

**Learning Objectives**

Upon completion of this activity, participants will be able to:

1. Distinguish the prevalence of sleep disturbance among Korean and Chinese American adults
2. Assess risk factors for sleep disturbance among Korean and Chinese American adults
3. Analyze how different forms of stress contribute to self-rated health
4. Evaluate how sleep disturbance might mediate the effects of stress on self-rated health

**Credit Hours —** 1.0

**EDITOR**

Ellen Taratus, MS, ELS

Editor

Preventing Chronic Disease

Atlanta, GA

**AUTHORS**

Brittany N. Morey, PhD, MPH

Department of Health, Society, and Behavior

Program in Public Health

University of California, Irvine

Irvine, California

Soomin Ryu, PhD, MA

Department of Epidemiology

School of Public Health

University of Michigan

Ann Arbor, Michigan

Yuxi Shi, MS

Department of Medicine

School of Medicine

University of California, Irvine

Irvine, California

Sunmin Lee, ScD, MPH

Department of Medicine

School of Medicine

University of California, Irvine

Irvine, California

**CME AUTHOR**

Charles P. Vega, MD

Health Sciences Clinical Professor of Family Medicine

University of California, Irvine School of Medicine

Charles P. Vega, MD, has the following relevant financial relationships:

Consultant or advisor for: GlaxoSmithKline; Johnson & Johnson Pharmaceutical Research & Development, LLC

## Introduction

Stress is a major contributor to health disparities among racial and ethnic populations in the US. Because of socioeconomic disadvantages and discriminatory experiences linked to historical and continued structural racism, racial and ethnic minority populations are more exposed to and susceptible to stress than are non-Hispanic White people (1,2). Asian, Black, and Hispanic or Latino populations in the US report higher levels of stress than the US non-Hispanic White population in several domains such as occupation, finances, childhood adversity, racial bias, and neighborhoods (1). Racial and ethnic differences in stress contribute to disparities in emotional strain, cardiovascular disease, and all-cause mortality (1,3). Research has focused on some of the pathways linking experiences of stress to health disparities among racial and ethnic minority populations, including allostatic load (cumulative burden of chronic stress) and maladaptive behaviors such as substance use and unhealthy diet (4,5). Less attention has been paid to the role of disturbed sleep to explain the relationship between stress and poor health.

This study focused on the mediating role of sleep disturbance in the association between stress and self-rated health among a sample of Chinese and Korean immigrants in the US. Self-rated health is a commonly used metric of overall health, and it has been applied in diverse populations, including immigrant Chinese and Korean Americans (6). Prior research provided empirical evidence that poor sleep mediated the associations between perceived stress and depression (7). Studies have additionally found disturbed sleep to mediate associations between stress and overall health and well-being among mothers and children experiencing trauma (8,9). Research suggests that many Asian Americans likely somaticize stressful experiences into physical symptoms such as sleep disturbances (10,11). Sleep disturbances lead to poor mental and physical functioning, including greater risk of inflammation, chronic diseases, and multimorbidity (12–14).

The current study contributes to this literature by examining the mediating role of sleep in the association between 3 types of stress (acculturative stress, perceived stress, and distress) and health. The sample consisted of Chinese and Korean immigrants, a group prone to experiencing these types of stress. Immigrants may experience acculturative stress — defined as the psychological impact, or stress reaction, of adapting to a new cultural context (15). Previous research suggests that acculturative stress is significantly associated with sleep disturbance or poor sleep quality among immigrant Chinese and Korean Americans (16). Furthermore, levels of perceived stress and reported distress may similarly be associated with poor sleep and subsequent poor health among Chinese and Korean immigrants in the US (6). To our knowledge, this is the first study to examine the role of sleep disturbance to explain the associations between stresses and health among Asians in the US. We first hypothesized that higher levels of acculturative stress, perceived stress, and distress would be associated with worse self-rated health. We also hypothesized that sleep disturbance would partially mediate the associations between stress and self-rated health.

## Methods

### Study sample

We used data from a randomized controlled trial to increase colorectal cancer screening among 400 Chinese and Korean Americans (200 Chinese and 200 Korean). Study participants were originally from China or Korea, aged 50 to 75 years, and living in the US for an average of 23 years. Participants were recruited in the Baltimore–Washington, DC, metropolitan area from primary care physicians’ clinics. The baseline survey data were collected from August 2018 through June 2020. Participants completed the survey either in person or by telephone in their preferred language (Mandarin, Korean, or English) after signing informed consent forms. Most (89%) participants completed a self-administered questionnaire in person; 11% of participants completed a research assistant–led telephone survey because of the COVID-19 outbreak in March 2020. This study was approved by the institutional review boards of the University of Maryland, College Park, and the University of California, Irvine.

### Measures

Self-rated health was the dependent variable, which was assessed by using the question “Would you say that in general your health is excellent, very good, good, fair, or poor?” Prior studies found self-rated health to be a valid measure of overall physical and mental health among Chinese and Korean populations in their respective languages (17,18). In this study, we used it as a continuous variable ranging from 1 (excellent) to 5 (poor), with higher scores indicating worse self-rated health.

Our independent variables of interest were 3 types of stress: acculturative stress, perceived stress, and distress. We assessed acculturative stress by using a 9-item scale from the National Latino and Asian American Study intended to measure stressors associated with the experience of being an immigrant in a US cultural and sociopolitical context; this scale has been widely used in the Chinese and Korean languages (15). Responses to the 9 items were dichotomous (yes = 1; no or not applicable = 0) and included the following: 1) feeling guilty for leaving family or friends in a home country, 2) receiving the same level of respect in the US as in a home country, 3) having limited contact with family or friends outside home country, 4) having difficulty in interactions with others because of English proficiency, 5) being treated badly because of speaking English poorly, 6) having difficulty in finding work because of Asian descent, 7) being questioned about legal status, 8) having concern about being deported if one were to go to a social or government agency, and 9) avoiding seeking health services due to fear of immigration officials. Item 2 was reverse-coded. We calculated acculturative stress as the sum of all 9 items (range, 0–9). Higher scores indicate greater acculturative stress.

We used a modified version of the Perceived Stress Scale to measure perceived stress (19). The Perceived Stress Scale has been validated in both the Chinese and Korean languages (20,21). This modified scale included 10 of the 14 items that measured self-reported stress over the past month: 1) how often have you been upset because of something that happened unexpectedly; 2) how often have you felt that you were unable to control the important things in your life; 3) how often have you felt nervous and stressed; 4) how often have you dealt successfully with irritating life hassles; 5) how often have you felt that you were effectively coping with important changes that were occurring in your life; 6) how often have you felt confident about your ability to handle your personal problems; 7) how often have you felt that things were going your way; 8) how often have you found that you could not cope with all the things that you had to do; 9) how often have you been able to control irritations in your life; 10) how often have you felt that you were on top of things. We coded each response on a range from 0 (“never”) to 4 (“very often”). Items 4, 5, 6, 7, 9, and 10 were reverse-coded. We calculated perceived stress as the sum of all 10 items (range, 0–40). Higher scores indicate greater perceived stress.

We measured distress by using a distress “thermometer” numbered from 0 at the bottom (“no distress”) to 10 at the top (“extreme distress”) (22). Respondents circled the number that best described how much distress they had been experiencing in the past week. Distress was a continuous variable (range, 0–10). Higher scores indicated greater distress.

Sleep disturbance was a potential mediator of the associations between stress and self-rated health. We assessed sleep disturbance by using the short-form version of the Sleep Disturbance Questionnaire from the Patient Reported Outcomes Measurement Information System (PROMIS), a validated and reliable measure of sleep disturbance (23). It included 8 items to measure self-reported perceptions of sleep quality, depth, and restoration during the past 7 days: 1) my sleep was restless; 2) I was satisfied with my sleep; 3) my sleep was refreshing; 4) I had difficulty falling asleep; 5) I had trouble staying asleep; 6) I had trouble sleeping; 7) I got enough sleep; and 8) my sleep quality was [very poor, poor, fair, good, or very good]. Respondents rated each item on a 5-point Likert scale, and we summed ratings to obtain a total raw score ranging from 8 to 40. Following the PROMIS guidelines for categorizing sleep disturbance, we converted the total raw score to a standardized T-score using conversion tables, with higher scores indicating greater sleep disturbances (23). Then, we recoded T-scores into a binary variable, with T-scores less than 55 indicating no or slight sleep disturbance and T-scores of 55 or more indicating mild, moderate, or severe sleep disturbance.

Sociodemographic characteristics included age (continuous years), sex (male or female), Asian subgroup (Chinese or Korean), marital status (married or cohabitating, or not currently married or cohabitating), education (less than high school, high school graduate or GED [General Educational Development], business or vocational school or some college, college graduate, or attended graduate or professional school), annual household income (<$20,000, $20,000–$39,999, $40,000–$59,999, $60,000–$79,999, $80,000–$99,999, or ≥$100,000), employment (full time, part time, or not employed), and health insurance status (private health insurance, Medicare or Medicaid, or no health insurance) based on self-report.

### Statistical analysis

First, we conducted a descriptive analysis for the sample overall and stratified by risk of sleep disturbance. We calculated means and SDs for all continuous variables, frequencies, and percentages for all categorical variables. To compare the differences between subgroups, we conducted 2-sample *t* tests for continuous variables and χ^2^ tests for categorical variables. Second, we used linear regression models to estimate associations between acculturative stress, perceived stress, distress, and self-rated health. We conducted 3 regression models for each exposure: Model 1 included the stress variable, adjusting for age; Model 2 added sex, Asian subgroup, marital status, education, annual household income, employment status, and health insurance status to Model 1; and Model 3 added sleep disturbance to Model 2. We then used the Karlson–Holm–Breen method (24) to conduct mediation analyses to estimate the degree to which sleep disturbance explained the association between stress and self-rated health. Using this method, we decomposed the total effect of stress on self-rated health into the direct (unmediated) effect of stress on self-rated health and indirect (mediated) effect of stress on self-rated health through sleep disturbance. This method also calculates percentages of the total effects of stress on self-rated health caused by sleep disturbance. We also created a simple conceptual model of the mediating role of sleep disturbance between stress and health.

We calculated these effects accounting for all sociodemographic covariates. We conducted analyses using Stata version 14 (StataCorp LLC).

## Results

Of the 400 participants, 327 (81.8%) had no or slight sleep disturbance, while 73 (18.2%) had mild, moderate, or severe sleep disturbance (Table 1). Participants with mild, moderate, or severe sleep disturbance had worse self-rated health and higher acculturative stress, perceived stress, and distress compared with participants with no or slight sleep disturbance. Additionally, participants with mild, moderate, or severe sleep disturbance were more likely to be female (vs male), Korean (vs Chinese), and not currently married or cohabitating (vs married or cohabitating) relative to participants with no or slight sleep disturbance.

**Table 1:** Characteristics of 400 Chinese and Korean Immigrants Aged 50 to 75 Years Recruited From Physicians’ Clinics in the Baltimore–Washington, DC, Metropolitan Area, August 2018–June 2020

Characteristic	Total (N = 400)	Sleep disturbance		*P* value
		None/slight	Mild/moderate/severe	
**No. (%) of participants**	400 (100.0)	327 (81.8)	73 (18.2)	—
**Self-rated health, mean (SD)^a^**	3.1 (1.0)	3.0 (1.0)	3.8 (0.9)	<.001
**Acculturative stress, mean (SD)^b^**	1.6 (1.5)	1.5 (1.5)	2.2 (1.8)	.002
**Perceived stress, mean (SD)^c^**	15.6 (4.3)	15.2 (4.3)	17.5 (3.9)	<.001
**Distress, mean (SD)^d^**	3.6 (2.4)	3.3 (2.3)	5.0 (2.4)	<.001
**Age, mean (SD), y**	58.4 (6.4)	58.4 (6.5)	58.4 (5.7)	.93
**Sex, n (%)**				
Female	211 (52.8)	163 (49.8)	48 (65.8)	.01
Male	189 (47.3)	164 (50.2)	25 (34.2)	
**Asian subgroup, n (%)**				
Chinese	200 (50.0)	173 (52.9)	27 (37.0)	.01
Korean	200 (50.0)	154 (47.1)	46 (63.0)	
**Marital status, n (%)**				
Not currently married or cohabitating	59 (14.8)	41 (12.5)	18 (24.7)	.008
Married/cohabiting	341 (85.3)	286 (87.5)	55 (75.3)	
**Education, n (%)**				
Less than high school	43 (10.8)	35 (10.7)	8 (11.0)	.22
High school graduate or GED	91 (22.8)	73 (22.3)	18 (24.7)	
Business/vocational school/some college	68 (17.0)	59 (18.0)	9 (12.3)	
College graduate	101 (25.3)	76 (23.2)	25 (34.2)	
Attended graduate/professional school	97 (24.3)	84 (25.7)	13 (17.8)	
**Household income, n (%), $**				
<20,000	62 (15.5)	46 (14.1)	16 (21.9)	.52
20,000–39,999	64 (16.0)	54 (16.5)	10 (13.7)	
40,000–59,999	85 (21.3)	68 (20.8)	17 (23.3)	
60,000–79,999	49 (12.3)	40 (12.2)	9 (12.3)	
80,000–99,999	32 (8.0)	26 (8.0)	6 (8.2)	
≥100,000	108 (27.0)	93 (28.4)	15 (20.5)	
**Employment status, n (%)**				
Working full time	231 (57.8)	190 (58.1)	41 (56.2)	.67
Working part time	84 (21.0)	66 (20.2)	18 (24.7)	
Not currently working	85 (21.3)	71 (21.7)	14 (19.2)	
**Health insurance status, n (%)**				
Private health insurance	243 (60.8)	200 (61.2)	43 (58.9)	.94
Medicare/Medicaid	74 (18.5)	60 (18.3)	14 (19.2)	
No health insurance	83 (20.8)	67 (20.5)	16 (21.9)	

Abbreviations: —, does not apply; GED, General Educational Development.

a Scale for self-rated health ranged from 1 (excellent) to 5 (poor).

b Scale consisted of 9 dichotomous (yes = 1; no or not applicable = 0) items. Scale ranged from 0 to 9, with higher scores indicating greater acculturative stress.

c A 10-item modified version of the Perceived Stress Scale (19) was used; scale ranged from 0 to 40, with higher scores indicating greater perceived stress.

d Measured by a distress “thermometer” numbered from 0 at the bottom (no distress) to 10 at the top (extreme distress). Respondents circled their response; scale ranged from 0 to 10, with higher scores indicating greater distress.

Linear regression models of the association between stresses and self-rated health, adjusting for covariates, showed that higher acculturative stress, perceived stress, and distress were associated with worse self-rated health across all models (Table 2). A 1-unit increase in acculturative stress was associated with worse self-rated health by 0.14 (95% CI, 0.07–0.20) in Model 1, which adjusted for age. This association was attenuated but still in the same direction when adjusted for covariates in Model 2. In other words, at the lowest acculturative stress level of 0, the predicted self-rated health was 2.9, and at the highest level of acculturative stress of 8.0, the predicted self-rated health was 4.1, accounting for sociodemographic characteristics. When sleep disturbance was added to Model 3, the association between acculturative stress and self-rated health was further attenuated, but still in the same direction. A 1-unit increase in perceived stress was associated with worse self-rated health by 0.08 (95% CI, 0.06–0.10) in Model 1, and the association was attenuated in Model 2. At the lowest perceived stress level of 2.0, the predicted self-rated health was 2.6, and at the highest perceived stress level of 26.0, the predicted self-rated health was 3.7, accounting for sociodemographic covariates. This association was slightly attenuated in Model 3 with the inclusion of sleep disturbance. Higher distress was also associated with worse self-rated health by 0.10 (95% CI, 0.06–0.14) and 0.11 (95% CI, 0.07–0.15) for every 1-unit increase in distress in Model 1 and Model 2, respectively. Accounting for sociodemographic factors, the predicted self-rated health was 2.8 at the lowest distress level of 0, and the predicted self-rated health was 3.8 at the highest distress level of 10.0. This association was attenuated, but still in the same direction accounting for sleep disturbance in Model 3. 

**Table 2:** Associations of Acculturative Stress, Perceived Stress, and Distress With Self-Rated Health^a^ in Linear Regression Analysis of Data From 400 Chinese and Korean Immigrants Aged 50 to 75 Years Recruited From Physicians’ Clinics in the Baltimore–Washington, DC, Metropolitan Area, August 2018–June 2020

Variable	Self-rated health to β (95% CI)								
	Acculturative stress^b^			Perceived stress^c^			Distress^d^		
	Model 1^e^	Model 2^f^	Model 3^g^	Model 1^e^	Model 2^f^	Model 3^g^	Model 1^e^	Model 2^f^	Model 3^g^
**Stress**	0.14 (0.07 to 0.20)	0.10 (0.03 to 0.16)	0.08 (0.01 to 0.14)	0.08 (0.06 to 0.10)	0.06 (0.04 to 0.09)	0.05 (0.03 to 0.08)	0.10 (0.06 to 0.14)	0.11 (0.07 to 0.15)	0.09 (0.05 to 0.13)
**Age**	0.01 (–0.01 to 0.03)	–0.01 (–0.03 to 0.01)	–0.01 (–0.02 to 0.01)	0.01 (–0.01 to 0.03)	0 (–0.02 to 0.02)	0 (–0.02 to 0.02)	0.01 (0 to 0.03)	0 (–0.02 to 0.01)	0 (–0.02 to 0.02)
**Sex**									
Female	—	0.22 (0.01 to 0.43)	0.16 (–0.04 to 0.37)	—	0.21 (0.01 to 0.42)	0.16 (–0.04 to 0.36)	—	0.19 (–0.01 to 0.40)	0.15 (–0.05 to 0.35)
Male	—	Reference	Reference	—	Reference	Reference	—	Reference	Reference
**Asian subgroup**									
Chinese	—	–0.21 (–0.43 to 0.02)	–0.14 (–0.36 to 0.07)	—	–0.14 (–0.36 to 0.08)	–0.09 (–0.31 to 0.12)	—	–0.21 (–0.43 to 0.01)	–0.16 (–0.38 to 0.05)
Korean	—	Reference	Reference	—	Reference	Reference	—	Reference	Reference
**Marital status**									
Not currently married	—	0.13 (–0.15 to 0.42)	0.07 (–0.21 to 0.34)	—	0.13 (–0.14 to 0.41)	0.07 (–0.20 to 0.34)	—	0.12 (–0.15 to 0.40)	0.07 (–0.20 to 0.34)
Married or cohabiting	—	Reference	Reference	—	Reference	Reference	—	Reference	Reference
**Education**									
Less than high school graduate	—	0.18 (–0.23 to 0.59)	0.19 (–0.21 to 0.59)	—	0.17 (–0.23 to 0.57)	0.18 (–0.21 to 0.57)	—	0.37 (–0.03 to 0.76)	0.34 (–0.06 to 0.73)
High school graduate or GED	—	0.35 (0.01 to 0.69)	0.36 (0.03 to 0.69)	—	0.35 (0.02 to 0.69)	0.36 (0.04 to 0.68)	—	0.51 (0.18 to 0.84)	0.49 (0.16 to 0.81)
Business/vocational school/some college	—	0.40 (0.06 to 0.74)	0.42 (0.09 to 0.76)	—	0.41 (0.08 to 0.75)	0.43 (0.11 to 0.76)	—	0.49 (0.15 to 0.82)	0.49 (0.16 to 0.82)
College graduate	—	0.35 (0.04 to 0.67)	0.32 (0.01 to 0.62)	—	0.33 (0.02 to 0.64)	0.30 (0 to 0.60)	—	0.42 (0.11 to 0.73)	0.38 (0.07 to 0.68)
Attended graduate/professional school	—	Reference	Reference	—	Reference	Reference	—	Reference	Reference
**Annual household income, $**									
<20,000	—	0.57 (0.17 to 0.98)	0.49 (0.09 to 0.88)	—	0.44 (0.04 to 0.84)	0.38 (–0.01 to 0.77)	—	0.57 (0.18 to 0.96)	0.50 (0.11 to 0.89)
20,000–39,999	—	0.44 (0.07 to 0.81)	0.46 (0.10 to 0.81)	—	0.28 (–0.09 to 0.64)	0.31 (–0.04 to 0.67)	—	0.40 (0.04 to 0.76)	0.42 (0.07 to 0.77)
40,000–59,999	—	0.22 (–0.11 to 0.55)	0.22 (–0.10 to 0.54)	—	0.17 (–0.16 to 0.49)	0.17 (–0.15 to 0.48)	—	0.18 (–0.14 to 0.50)	0.18 (–0.13 to 0.50)
60,000–79,999	—	0.08 (–0.30 to 0.46)	0.08 (–0.29 to 0.45)	—	0.01 (–0.37 to 0.38)	0.01 (–0.35 to 0.38)	—	0.01 (–0.36 to 0.38)	0.02 (–0.35 to 0.38)
80,000–99,999	—	–0.10 (–0.52 to 0.32)	–0.10 (–0.51 to 0.30)	—	–0.23 (–0.64 to 0.19)	–0.21 (–0.62 to 0.19)	—	–0.19 (–0.59 to 0.22)	–0.17 (–0.57 to 0.23)
≥100,000	—	Reference	Reference	—	Reference	Reference	—	Reference	Reference
**Employment status**									
Working part time	—	–0.01 (–0.28 to 0.26)	0 (–0.26 to 0.26)	—	0.07 (–0.20 to 0.33)	0.07 (–0.19 to 0.32)	—	0.07 (–0.19 to 0.33)	0.06 (–0.19 to 0.32)
Not currently working	—	0.05 (–0.24 to 0.34)	0.09 (–0.19 to 0.37)	—	0.06 (–0.22 to 0.34)	0.09 (–0.18 to 0.37)	—	0.11 (–0.17 to 0.39)	0.13 (–0.15 to 0.40)
Working full time	—	Reference	Reference	—	Reference	Reference	—	Reference	Reference
**Health insurance status**									
Medicare/Medicaid	—	–0.05 (–0.37 to 0.26)	–0.04 (–0.35 to 0.26)	—	–0.10 (–0.40 to 0.21)	–0.08 (–0.38 to 0.22)	—	–0.13 (–0.44 to 0.18)	–0.11 (–0.41 to 0.19)
No health insurance	—	0.11 (–0.16 to 0.37)	0.11 (–0.15 to 0.37)	—	0.10 (–0.16 to 0.37)	0.10 (–0.15 to 0.36)	—	0.12 (–0.13 to 0.38)	0.12 (–0.13 to 0.38)
Private health insurance	—	Reference	Reference	—	Reference	Reference	—	Reference	Reference
**Sleep disturbance**									
Mild, moderate, or severe	—	—	0.61 (0.36 to 0.86)	—	—	0.55 (0.30 to 0.80)	—	—	0.49 (0.24 to 0.75)
None to slight	—	—	Reference	—	—	Reference	—	—	Reference

a Scale for self-rated health ranged from 1 (excellent) to 5 (poor).

b Scale consisted of 9 dichotomous (yes = 1; no or not applicable = 0) items. Scale ranged from 0 to 9, with higher scores indicating greater acculturative stress.

c A 10-item modified version of the Perceived Stress Scale (19) was used; scale ranged from 0 to 40, with higher scores indicating greater perceived stress.

d Measured by a distress “thermometer” numbered from 0 at the bottom (no distress) to 10 at the top (extreme distress). Respondents circled their response; scale ranged from 0 to 10, with higher scores indicating greater distress.

e Model 1: Stress + age.

f Model 2: Model 1 + sex, Asian subgroup, marital status, education, household income, employment status, health insurance status.

g Model 3: Model 2 + sleep disturbance.

The mediation analyses using the Karlson–Holm–Breen method showed the total effects of each stress on self-rated health, the decomposed direct (unmediated) effects of each stress on self-rated health, and the indirect (mediated) effects of each stress on self-rated health through sleep disturbance, accounting for covariates (Table 3). The total effect of acculturative stress on self-rated health was 0.10. A direct effect of acculturative stress on self-rated health (β = 0.08) remained independent of the potential mediator. The indirect effect of acculturative stress on self-rated health through sleep disturbance was 0.02, and 21.7% of total effect was due to sleep disturbance alone accounting for all covariates. The total and direct effects of perceived stress on self-rated health were 0.06 and 0.05, while the indirect effect mediated by sleep disturbance was 0.01. Sleep disturbance accounted for 14.9% of the total effect of perceived stress on self-rated health. For the association between distress and self-rated health, the total, direct, and indirect effects were 0.11, 0.09, and 0.02, respectively. Sleep disturbance accounted for 18.7% of the total effect of distress on self-rated health. A conceptual model (Figure) visually outlines the various relationships.

**Table 3:** Sleep Disturbance Mediating the Association Between Stresses and Self-Rated Health Among 400 Chinese and Korean Immigrants Aged 50 to 75 Years Recruited From Physicians’ Clinics in the Baltimore–Washington, DC, Metropolitan Area, August 2018–June 2020^a^

Decomposition of effects	β (SE)	*P* value	Percentage of total effect due to sleep disturbance
**Total effect of acculturative stress on self-rated health**	0.10 (0.03)	.002	21.6
Direct (unmediated) effect of acculturative stress on self-rated health	0.08 (0.03)	.02	
Indirect (mediated) effect of acculturative stress on self-rated health through sleep disturbance	0.02 (0.01)	.02	

**Total effect of perceived stress on self-rated health**	0.06 (0.01)	<.001	14.9
Direct (unmediated) effect of perceived stress on self-rated health	0.05 (0.01)	<.001	
Indirect (mediated) effect of perceived stress on self-rated health through sleep disturbance	0.01 (0)	.005	

**Total effect of distress on self-rated health**	0.11 (0.02)	<.001	18.7
Direct (unmediated) effect of distress on self-rated health	0.09 (0.02)	<.001	
Indirect (mediated) effect of distress on self-rated health through sleep disturbance	0.02 (0.01)	.002	

a All effects were calculated by accounting for the following covariates: age, sex, Asian subgroup, marital status, education, household income, employment status, and health insurance status.

**Figure:**
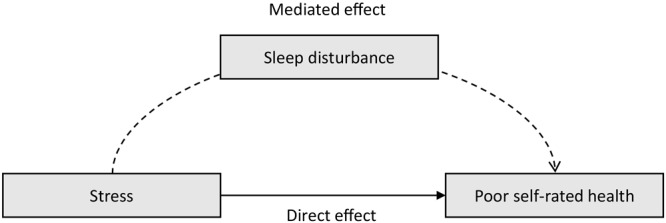
Conceptual model of the mediating role of sleep disturbance between stress and self-rated health.

## Discussion

This study tested the mediating role of sleep in the relationship between 3 types of stress and health. Specifically, we studied whether acculturative stress, perceived stress, and distress were associated with self-rated health, and whether this could be partly explained by higher stress increasing the odds of having sleep disturbance and higher sleep disturbance contributing to worse self-rated health. The results indicated that higher levels of acculturative stress, perceived stress, and distress were associated with worse self-rated health, which supported our first hypothesis. We also found that sleep disturbance was a partial mediator of these associations, supporting the second hypothesis. Sleep disturbance explained 21.7%, 14.9%, and 18.7% of the associations of acculturative stress, perceived stress, and distress with self-rated health, respectively. We evaluated the 3 types of stress separately in this analysis, because they likely overlap and influence one another. For example, people with high levels of acculturative stress likely experience and report more distress as well. Nevertheless, it is notable that all 3 forms of stress were associated with self-rated health, and sleep disturbance was a mediator for all stress types to slightly different degrees.

Our findings align with previous empirical evidence that sleep quality was a mediator between stress and poor health outcomes. Lee and Hsu found that poor sleep quality mediated the association between stress and poor mental health among US mothers of infants with a low birth weight (8). Similarly, Steffen and Bowden showed that sleep quality mediated the relationship between perceived racism and depression among US Latinos (25). In China, a study demonstrated that the association between perceived stress and depression was partly mediated by sleep quality among older populations (7). A review suggested evidence for sleep’s mediating role in the relationship between traumatic stress and health outcomes among people in the US who experienced specific adverse life events such as HIV diagnosis, war, hurricane, or death of a spouse (9). Our results indicated that acculturative stress, perceived stress, and distress functioned in similar ways to other chronic or traumatic stress in contributing to worse health via sleep disturbance among Chinese and Korean immigrants in the US. Sleep disturbance is a salient intermediary between stress and other health outcomes, potentially explaining one-fifth of this relationship. Therefore, sleep disturbance may be a signal to health providers of underlying experiences of stress and potential for future worsened health. In 2022, the American Heart Association added healthy sleep to its checklist of important health and lifestyle factors for cardiovascular health (26). Future interventions to lower stress, and subsequently, promote sleep hygiene could be considered to prevent cardiovascular disease for racial and ethnic minority populations.

Herein we presented a framework for the mediating role of sleep linking stress to health. First, stress could predispose people to sleep disturbances by stimulating the hypothalamic-pituitary-adrenal axis to release attention- and arousal-related hormones such as cortisol, noradrenaline, and adrenaline as part of the sympathetic nervous system’s fight-or-flight response (27). These hormones interfere with the body’s ability to maintain quality sleep (27). Poor sleep health may then result in risk of inflammatory disease by increasing the levels of C-reactive protein and interleukin-6 (12). Another mechanism linking poor sleep to disease risk is decreased serotonin, a neurotransmitter that regulates normal circadian rhythms and is also at low levels in people with depression (14). These mechanisms link stress to poor sleep, and poor sleep to worse health outcomes, including poor physical functioning, depression, and chronic disease (13,14).

To our knowledge, ours is the first study to demonstrate the mediating role of sleep in the association between stress and health among a sample of Asian Americans. Although Asian Americans are the fastest-growing racial and ethnic group in the US (28), they are less represented than other racial groups in stress-related research. This may be because Asian Americans have historically been stereotyped as a “model minority” whose perceived success in the US leads to the incorrect assumption that they do not experience stress caused by discrimination or low socioeconomic status (11). This myth obscures the struggles of many Asian Americans, especially those who have low incomes. Currently, Asians in the US have the largest income gap of any racial group in the country, with the top 10% earning more than 6 times that of the bottom 10% of Asian Americans (3). Furthermore, Asian Americans are extremely diverse, representing people from more than 50 countries, and many subgroups encounter stressful events that do not gain adequate attention. Asian Americans have been depicted as perpetual foreigners and outsiders while experiencing racial discrimination and pressure to conform to the model minority myth (11). Moreover, two-thirds of Asian Americans are non–US born, which exposes them to unique stressors such as acculturative stress (28). When experiencing several stressful challenges, Asian Americans may have difficulty in coping, which manifests in sleep disturbance. The few studies on sleep health for this racial minority group have shown that Asian Americans are more likely to report short sleep duration, greater daytime sleepiness, and have more sleep disordered breathing than White populations in the US (29,30). Among Asian Americans, experiences of racial discrimination and acculturative stress have been associated with greater sleep disturbance (10,16). Notably, the prevalence of sleep disturbance in our sample of Chinese and Korean Americans was similar to that found in the general US population (23). Sleep disturbance may be an even greater problem among Asian American subgroups experiencing heightened levels of stress.

Although our study demonstrates novel findings, we have the following limitations to highlight. First, the cross-sectional nature of the data set did not enable us to establish causal mechanisms. Associations may be in the other direction: worse self-rated health may lead to sleep disturbance, which then increases stress. Therefore, our findings on the mediating role of sleep should be considered preliminary. Nevertheless, our proposed mechanism aligns with previous longitudinal work linking stress to sleep and sleep to health outcomes (10,12–14). Second, our study used a unique sample of Chinese and Korean immigrants aged 50 to 75 years living in the Baltimore–Washington, DC, metropolitan area. Our findings are not generalizable to all Asian Americans or to other immigrant groups. The middle-aged and older adults in our study sample were likely experiencing more health issues related to aging and have different types of life stressors than younger people. To improve generalizability, future studies should include both US-born and non–US-born populations in a broader age range who are from diverse racial and ethnic backgrounds. Furthermore, more research that includes other disaggregated Asian subgroups who experience other types or levels of stress is needed. Last, we used only a single retrospective (ie, during the past 7 days) self-reported measure to assess sleep disturbance. This measure may not be the most accurate measure although it is widely used, valid, and reliable in diverse populations. Future studies could use other validated, objective measures of sleep disturbance, such as actigraphy, to confirm these findings.

Despite some limitations, our study has crucial implications for preventing chronic disease. There is a need to design and implement intervention programs tailored for Asian Americans and other racial and ethnic minority populations that could reduce stress and address sleep disturbance, which are significant risk factors for health. For example, clinicians could develop therapeutic interventions to bolster protective factors that mitigate stress and sleep disorders among Asian immigrants. These interventions can offer mental health or behavioral health services that provide patients with tools to manage stress and improve sleep hygiene. Sleep health can be an important focus for prevention-oriented interventions given the current findings that sleep disturbance is a symptom of stress that has a strong link to self-rated health. Future research could examine whether improving sleep health promotes resilience and buffers against the negative effects of stress on health among racial and ethnic minority populations experiencing heightened stress.

## Post-Test Information

To obtain credit, you should first read the journal article. After reading the article, you should be able to answer the following, related, multiple-choice questions. To complete the questions (with a minimum 75% passing score) and earn continuing medical education (CME) credit, please go to http://www.medscape.org/journal/pcd. Credit cannot be obtained for tests completed on paper, although you may use the worksheet below to keep a record of your answers.

You must be a registered user on http://www.medscape.org. If you are not registered on http://www.medscape.org, please click on the “Register” link on the right hand side of the website.

Only one answer is correct for each question. Once you successfully answer all post-test questions, you will be able to view and/or print your certificate. For questions regarding this activity, contact the accredited provider, CME@medscape.net. For technical assistance, contact CME@medscape.net. American Medical Association’s Physician’s Recognition Award (AMA PRA) credits are accepted in the US as evidence of participation in CME activities. For further information on this award, please go to https://www.ama-assn.org. The AMA has determined that physicians not licensed in the US who participate in this CME activity are eligible for AMA PRA Category 1 Credits™. Through agreements that the AMA has made with agencies in some countries, AMA PRA credit may be acceptable as evidence of participation in CME activities. If you are not licensed in the US, please complete the questions online, print the AMA PRA CME credit certificate, and present it to your national medical association for review.

## Post-Test Questions

### Study Title: The Mediating Role of Sleep Disturbance on the Association Between Stress and Self-Rated Health Among Chinese and Korean Immigrant Americans

#### CME Questions

1. What was the rate of sleep disturbance in the current study by Morey and colleagues among Korean and Chinese American adults?
  - 6%
  - 18%
  - 31%
  - 55%
2. Which one of the following variables was associated with a higher rate of sleep disturbance in the current study?
  - Being a male
  - Lower income level
  - Being Chinese American
  - Not being married
3. Which one of the following variables was associated with worse self-rated health (SRH) in the current study?
  - Distress only
  - Distress and perceived stress only
  - Distress and acculturative stress only
  - Distress, perceived stress, and acculturative stress
4. Sleep disturbance mediated approximately what percentage of the effects of stress on SRH in the current study?
  - Sleep disturbance did not significantly mediate effects on SRH
  - 5% to 10%
  - 15% to 20%
  - 40% to 45%
